# Electron Beam Crosslinking as a Strategy for the Synthesis of Pectin/PVA Aerogels: Effect of Concentration and Radiation Dose

**DOI:** 10.3390/ijms27188047

**Published:** 2026-09-10

**Authors:** Dawid Szymborski, Radosław Wach, Pavel Gurikov, Anna Strąkowska, Krzysztof Strzelec, Maher Saifi, Renata Boris, Agnė Kairytė

**Affiliations:** 1 Institute of Polymer and Dye Technology, Faculty of Chemistry, Lodz University of Technology, Stefanowskiego 16, 90-537 Lodz, Poland; 2 Institute of Applied Radiation Chemistry, Faculty of Chemistry, Lodz University of Technology, Wroblewskiego 15, 93-590 Lodz, Poland; 3 Institute of Thermal Separation Processes, Hamburg University of Technology, Eißendorfer Straße 38, 21073 Hamburg, Germany; 4 Aerogel-it GmbH, Albert-Einstein-Str. 1, 49076 Osnabruck, Germany; 5 United Nations University Hub on Engineering to Face Climate Change at the Hamburg University of Technology, United Nations University, Institute for Water, Environment and Health (UNU-INWEH), Hamburg, Eißendorfer Straße 38, 21073 Hamburg, Germany; 6 Aix Marseille University, CNRS, Centrale Marseille, M2P2 UMR 7340, 13451 Marseille, France; 7 Laboratory of Composite Materials, Institute of Building Materials, Faculty of Civil Engineering, Vilnius Gediminas Technical University, Linkmenų St. 28, 08217 Vilnius, Lithuania

**Keywords:** polysaccharide, mesoporous, pectin, poly(vinyl alcohol), aerogels, supercritical CO_2_, ionizing radiation, radiation crosslinking, shrinkage control, biopolymer

## Abstract

Radiation techniques represent an innovative and alternative method for polymer gelation or crosslinking; their application in aerogel research has not been extensively investigated. The irradiation of polymer solutions, either with or without a crosslinking agent, may result in stable polymer gels with a highly developed mesoporous structure. In this study, the effect of the pectin polysaccharide concentration, the presence of PVA, and the dose of ionizing radiation (27–58 kGy) were systematically investigated, particularly with respect to the structural, textural, and mechanical properties. The pectin/PVA and PVA were crosslinked in aqueous solution via ionizing radiation using an electron beam. The results demonstrated that the radiation crosslinking of a hydrophilic synthetic polymer in the presence of natural polysaccharides, such as pectin, is an effective strategy for engineering porous materials with a controlled structure, namely aerogels, of good stability, and favorable functional properties. The resulting composite aerogels were characterized by a BET-determined specific surface area of 200–241 m^2^/g and an estimated porosity of over 91%, compared with values of 28–46 m^2^/g, 0.22 g/cm^3^ and approximately 81.5% for aerogels produced using the freeze–thaw method.

## 1. Introduction

In 1932, Samuel Kistler was the first who reported a material in which the liquid was replaced by air, thereby producing a material characterized by a particularly low density and high porosity with a high specific surface area. This pioneering discovery initiated a research trajectory that focused on synthesizing materials with diverse properties and applications. Now, aerogels are the most porous materials known and they are obtained from gels where the liquid medium is replaced by gas, and the removal of bulking agents does not compromise their properties or structure. One of their most unique properties is their very low thermal conductivity, for instance the aerogel from silica, having >90% porosity with a thermal conductivity of 12–20 mW·m^−1^·K^−1^, which is greatly lesser than the conductivity of regular insulation materials [1].

Originally, aerogels were primarily synthesized from inorganic materials, such as silica. Nevertheless, their fragility and brittleness have limited their wider application, especially in dynamic or heavy-duty environments [2,3]. Recent developments in the field of bio-based aerogels have highlighted the potential of combining polyvinyl alcohol (PVA) with natural polymers such as chitosan, alginate, or lignin to enhance biodegradability and environmental sustainability without compromising mechanical integrity or porosity. Yang et al. demonstrated that lignin nanoparticles incorporated into PVA/chitosan hydrogels confer antioxidant and antibacterial properties whilst maintaining the integrity of the network [4], whilst Zhang et al. developed a composite hydrogel based on lignin, chitosan, and PVA for use in wound dressings [5]. A more comprehensive review of lignin-based hydrogel systems and their biomedical applications was presented by Hachimi Alaoui et al. [6], whilst Okutucu [7] focused specifically on bio-based aerogels. Seifi et al. demonstrated that dual-network hydrogels of chitosan, alginate, and PVA, reinforced with carbon nanomaterials, can serve as biomimetic scaffolds for bone regeneration [8].

Consequently, research efforts are focused on developing aerogels from various materials. Polyvinyl alcohol is an example of a hydrophilic, water-soluble polymer characterized by low toxicity and biocompatibility that may be an interesting alternative for aerogels. Furthermore, it contains hydroxyl groups that may participate in various reactions, making the polymer easy to functionalize in order to enhance its properties [9,10]. Several successful attempts have been made to develop a PVA aerogel. For instance, the freeze–thaw method was employed to produce a thin, stable gel film, which was subsequently dried in supercritical carbon dioxide [9]. The freeze–thawing method leads to the formation of physical crosslinking and therefore results in a physical hydrogel. However, the freezing of an aqueous PVA solution limits the spatial movement of long polymer chains and allows hydroxyl groups to form hydrogen bonds. Subsequent thawing of the sample restores the mobility of the polymer chains, resulting in a rearrangement of the structure and bringing successive hydroxyl groups closer together to form additional hydrogen bonds in the next freezing cycle.

The literature still lacks reports on obtaining a PVA aerogel with a defined specific surface area using the freeze–thaw method. An often discussed challenge in this approach is the influence of the ice crystal morphology on the resulting pore architecture, which varies depending on the cooling rate, initial polymer concentration, and presence of additives, particularly making nucleation centers [11]. Freeze–thaw cycles, while a simple and non-toxic method (as it does not involve initiators or crosslinkers), typically lead to an inhomogeneous structure, particularly in bulky samples where heat transfer is limited [12]. Furthermore, emerging fabrication strategies such as 3D printing of aerogel inks and the direct ink writing (DIW) of PVA-based precursors offer novel ways to control the aerogel architecture at multiple length scales [13,14]. This allows the development of anisotropic structures with tunable mechanical and thermal behavior, which are promising for wearable electronics and thermal insulation layers, for instance in aerospace applications [14,15].

In recent years, several reports have focused on pectin aerogels [16,17,18]. Pectins are complex polysaccharides and oligosaccharides comprising mainly D-galacturonic acid residues linked by α-1,4 glycosidic bonds, partially esterified with methyl groups. The synthesis of aerogels from these natural materials has been accomplished through the utilization of crosslinking initiated by a pH change or involving divalent calcium cations. The resulting materials have been found to exhibit a high specific surface area (400–600 m^2^/g), and their primary applications have centered on the domain of drug delivery systems [18].

The use of radiation to initiate crosslinking and gel formation, whether with or without a crosslinking agent, is efficient and rapid, as it may take only a few seconds or minutes. Nevertheless, achieving the crosslinking of pectin in aqueous solutions of moderate concentrations (<10%) requires a crosslinking agent. Functionalized polysaccharides can undergo crosslinking, forming a network structure after irradiation in an aqueous solution. It was demonstrated that the crosslinking of some polysaccharides, especially without crosslinking agents, is possible with radiation [19]. However, the efficiency of such crosslinking is too low to produce a gel stable enough that it can subsequently undergo solvent exchange and drying in supercritical carbon dioxide. It is therefore feasible to use a crosslinking agent, as in the case of carboxymethyl chitosan [20], or employ internal unsaturated bonds capable of polymerization, which has been demonstrated, for instance, for dextran methacrylate [21]. An alternative route may be to admixture a synthetic hydrophilic polymer capable for generating active radicals prone to recombination, also involving radicals, in a polysaccharide; one example is poly(vinyl alcohol).

The irradiation of PVA in an aqueous solution [22,23] leads, through transient products of water radiolysis, to the formation of free radicals on macromolecules that participate in the crosslinking of PVA, resulting in stable hydrogels [24,25]. The advantage of this method is that it does not require the use of crosslinking agents, which are often toxic, as compounds bearing unsaturated bonds are highly reactive. Radiation engineering, specifically polymer crosslinking, is used to produce wound dressings or scaffolds for drug delivery, among other applications [26,27]. Radiation-induced crosslinking also offers greater control over the network density and crosslinking homogeneity, especially when processing large-volume samples.

Drying techniques also play a crucial role in governing the microstructure and other properties, determining the applications of PVA aerogels. While supercritical CO_2_ drying is widely regarded as the gold standard for preserving the aerogel structure, it is energy-intensive and costly. The emphasis on sustainable production methods reflects a broader commitment to green chemistry principles, driven not only by environmental trends but also by urgent global challenges demanding eco-friendly solutions in advanced material manufacturing. In contrast, freeze-drying is more accessible, but can lead to pore collapse due to sublimation-induced stress [28].

The objective of this study was to develop and characterize aerogels from pectin and polyvinyl alcohol. To the best of our knowledge, this is the first report in which ionizing radiation is used to fix the structure of a hydrogel as a deliberate step preceding supercritical CO_2_ drying, that is, as a route to aerogels; radiation techniques have so far been applied to hydrogel synthesis but not as a structure-control tool in aerogel technology. The hypothesis was formulated that ionizing radiation could crosslink pectin and polyvinyl alcohol, thereby forming a hydrogel that could subsequently be processed through supercritical drying. This was compared with aerogels produced by solidifying a PVA solution through freeze–thaw cycles. A series of experimental studies was conducted with the objective of describing the structure of the resulting products.

## 2. Results

The gelling ability of these composites is shown in Table 1, and the effect of the PVA weight fraction on hydrogel formation is shown.

Stable gels were obtained for the PVA/P/1 and PVA/P/2 samples at every radiation dose, whereas the PVA/P/3 and PVA/P/4 samples, containing a lower amount of PVA, did not yield sufficiently stable gels to undergo further processing, i.e., solvent exchange and drying with a supercritical medium. For doses below 25 kGy, no formation of a stable gel was observed, which could then be converted into an aerogel. This is due to the PVA concentration being too low for a stable polymer network capable of maintaining the gel structure. The study also attempted to crosslink polyvinyl alcohol without other additives. Although a stable gel was obtained (sample PVA/1 (27, 41, 58) kGy), the samples shrank during drying under supercritical carbon dioxide conditions that caused termination of investigations on sole PVA aerogels.

### 2.1. Morphological Analysis

**Figure 1:**
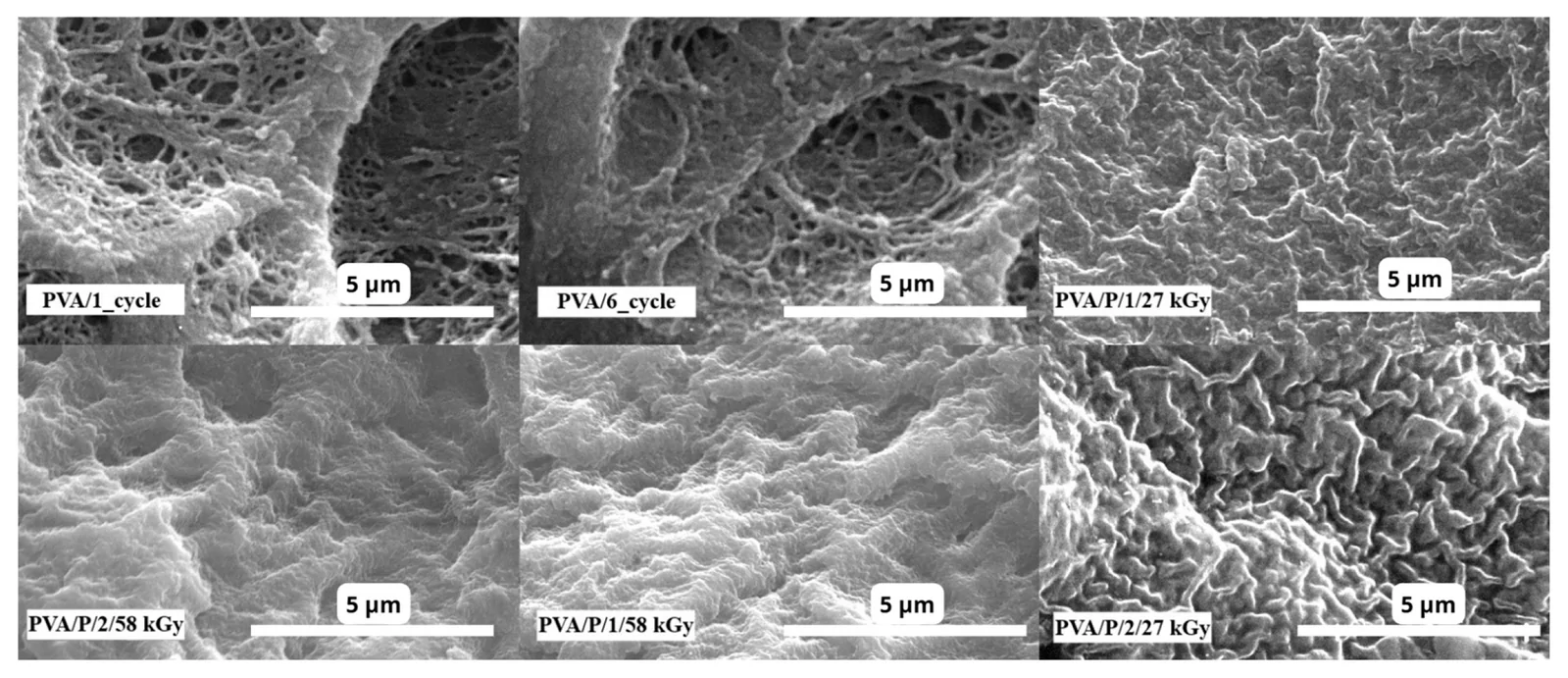
Scanning electron microscopy images for PVA and PVA/pectin samples crosslinked via freeze–thaw cycles (PVA) and with electron beam irradiation (PVA/P).

Scanning electron microscopy was used to evaluate the structure of the pectin/PVA and PVA-based aerogels. The test was carried out on approximately 20% of the samples, which were selected at random from each composition. To expose the internal structure of the samples, they were broken open—the morphology of representative specimens is visualized in Figure 1. The morphology depends on the crosslinking technique used. The structure of the PVA network formed as a result of freezing and thawing cycles differs from that of radiation-crosslinked samples. Open pores of the material are visible. In the case of radiation-crosslinked samples, there are, in principle, no open pores in the material. Lower irradiation doses result in greater surface development at the micrometer level, but less than for freeze–thaw samples.

### 2.2. Physical Properties

The specific surface area of the pectin and polyvinyl alcohol composite, measured using the BET method, is similar for all compositions of approximately 220 m^2^/g (Table 2). In contrast, the samples crosslinked via the freeze–thaw process have a low specific surface area, averaging 33 m^2^/g. This result shows that the electron beam crosslinking of polyvinyl alcohol and pectin composites leads to higher specific surface areas of resulting aerogels, which is due to the presence of a smaller number of macropores in the radiation-crosslinked samples. An increase in the surface area of radiation-crosslinked samples suggests the formation of a subtle porous network, potentially due to more uniform/random and/or efficient crosslinking at the molecular level. This process may limit polymer chain aggregation, preserving smaller pore dimensions and increasing the accessible internal surface. On the other hand, hydrogels obtained via freezing and thawing are macroporous due to the unrestricted growth of ice crystals. The specific surface area decreases with an increasing number of cycles.

**Figure 2:**
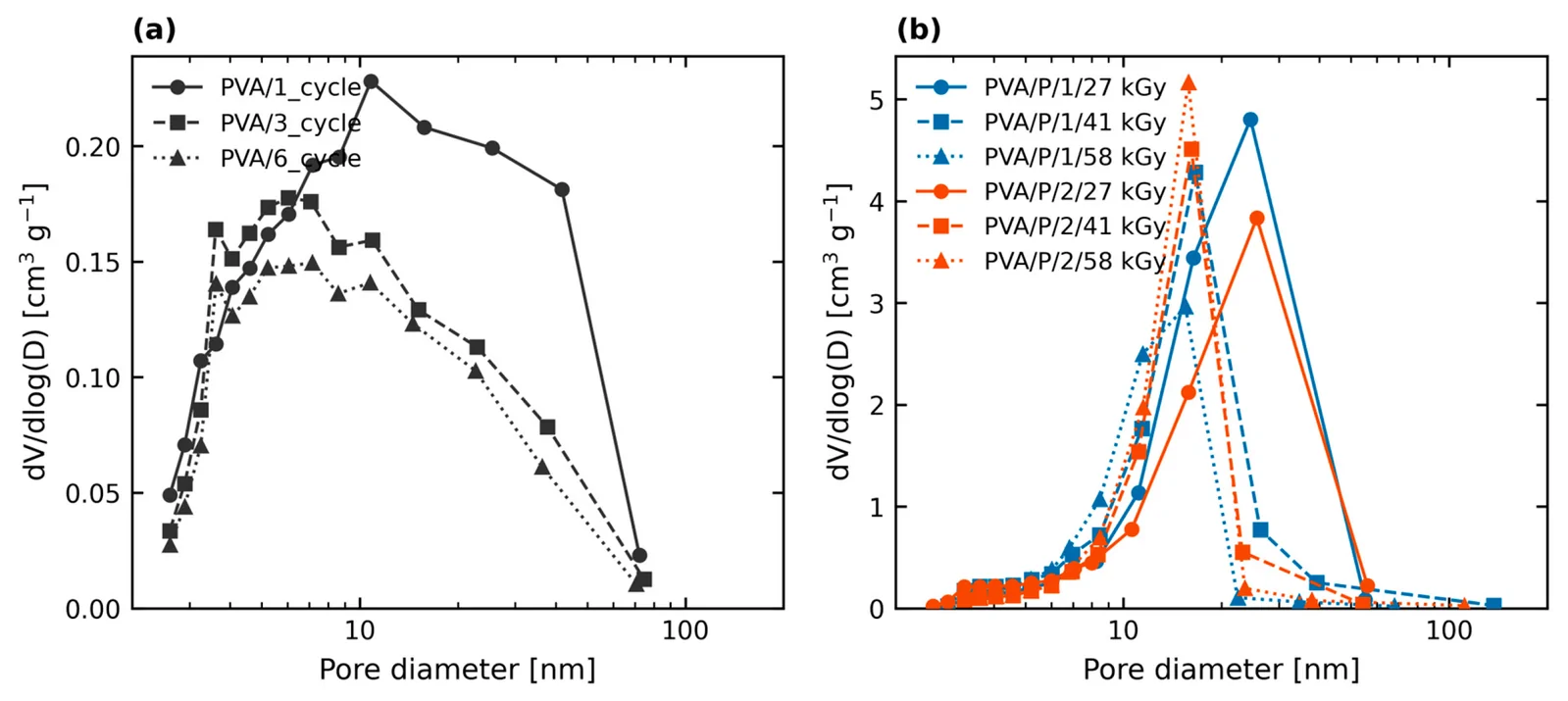
BJH pore size distribution for (**a**) PVA samples crosslinked with FT. (**b**) PVA/P/1 and 2 samples crosslinked with EB.

**Figure 3:**
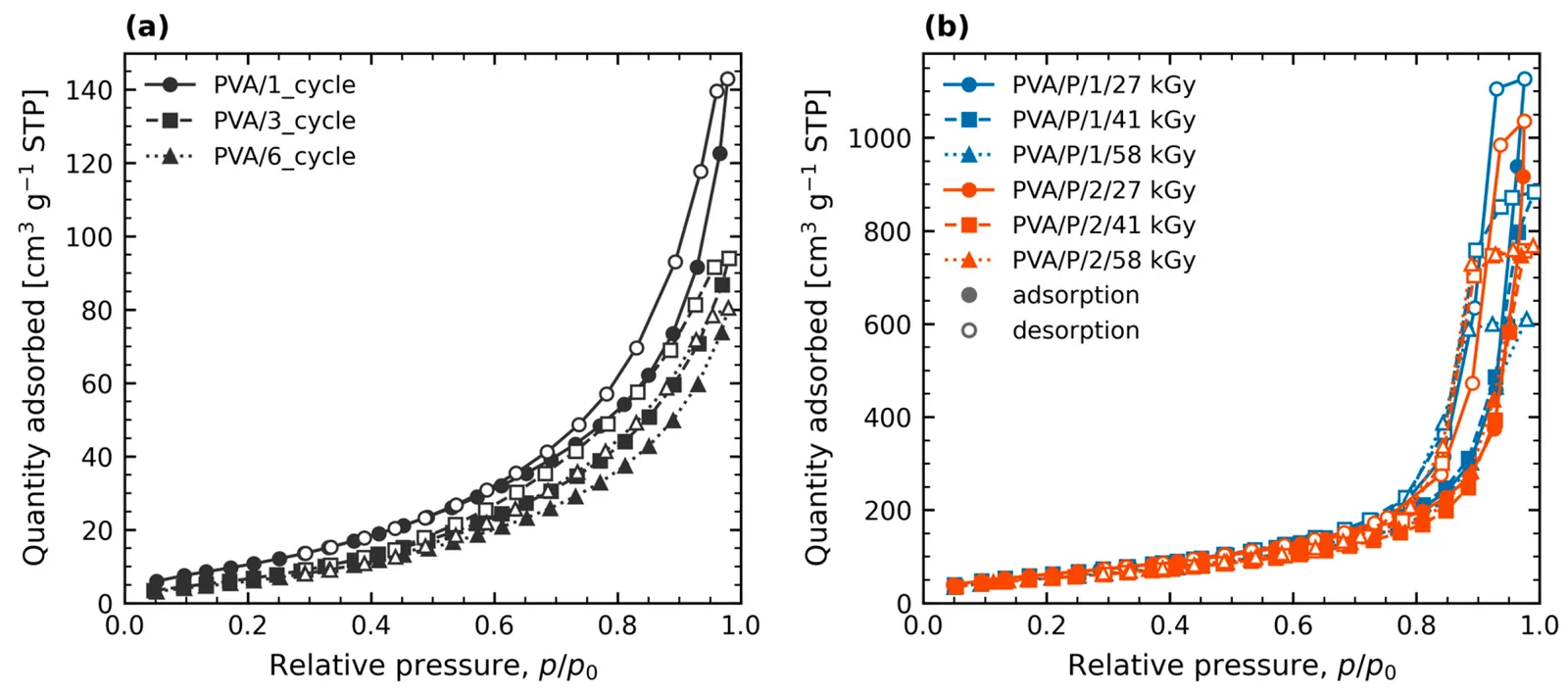
Adsorption–desorption isotherms for (**a**) PVA samples crosslinked with FT. (**b**) PVA/P/1 and 2 samples crosslinked with EB.

Table 2 presents the bulk density of aerogels. Samples crosslinked with an electron beam are characterized by an average bulk density of approximately 0.1 g/cm^3^, while samples crosslinked with freeze–thaw cycles are characterized by a density of 0.22 g/cm^3^. Given that the skeletal density of PVA is 1.19 g/cm^3^, the theoretical density of an aerogel derived from a 10 wt. % PVA solution would be approximately 0.119 g/cm^3^ (assuming the density of the aqueous solution is that of pure water). The experimental value of 0.22 g/cm^3^ indicates that approximately 55% of the volume is retained after solvent exchange and drying. Considering the BET results, it can be concluded that crosslinking with freeze–thaw cycles promotes the formation of a structure containing a large fraction of macropores generated via ice crystal templating. The samples are characterized by a hierarchical porosity, comprising both smaller and larger pores in the mesoporous range, as well as macropores, as demonstrated via scanning electron microscopy (SEM) (Figure 1), particularly in the samples crosslinked via freeze–thaw cycles (Figure 2a). In contrast, the samples crosslinked using an electron beam (Figure 2b) were characterized by a higher proportion of smaller pores in the range of (1–10 nm). This is due to the absence of freeze–thaw cycles. The complete nitrogen-adsorption and desorption isotherms are shown in (Figure 3). All samples exhibit Type IV(a) isotherms with a hysteresis loop, confirming that the adsorption process is governed via capillary condensation in the mesopores, combined with an unlimited increase near saturation, reflecting the additional contribution of macropores and interparticle voids. However, the two crosslinking methods differ in terms of all quantitative characteristics of the isotherm. Electron-beam-crosslinked aerogels adsorb 610–1127 cm^3^ g^−1^ at saturation, whereas materials subjected to the freeze–thaw process adsorb 81–143 cm^3^ g^−1^ under STP conditions; even in the monolayer region (p/p_0_ < 0.1), their adsorption capacity is approximately one order of magnitude higher, in direct proportion to the BET surface areas listed in Table 2.

To better quantify the structural differences between aerogels obtained via freeze–thaw and electron-beam crosslinking, total porosity was estimated based on bulk and skeletal densities. The porosity values ranged from approximately 81 to 82% for freeze–thaw-crosslinked PVA aerogels and exceeded 91% for electron-beam-crosslinked PVA/pectin composites. These findings confirm that EB crosslinking enables the preservation of a substantially more porous structure during supercritical drying. Interestingly, despite the moderate shrinkage observed for irradiated samples, their porosity remained significantly higher than that of freeze–thaw-derived aerogels, suggesting that macroscopic shrinkage does not necessarily correspond to a loss of nanoscale porosity. This observation is consistent with the significantly higher BET surface area and reduced crystallinity observed for irradiated samples. It is hypothesized that radiation-induced crosslinking suppresses PVA chain ordering and limits network densification during solvent exchange and supercritical drying, thus preserving a more developed mesoporous architecture. In contrast, freeze–thaw processing leads predominantly to macroporous structures due to unrestricted ice crystal growth, resulting in a lower specific surface area despite comparatively lower total shrinkage.

Table 3 illustrates the shrinkage that occurred during the solvent exchange and the drying processes. The samples of PVA (crosslinked with freeze–thawing) underwent substantial shrinking during drying, with a volume change of more than 30% being typical, confirming what is reflected by the density. This value is comparatively higher concerning other materials subjected to supercritical conditions during the drying process. An interesting phenomenon is the low shrinkage of the samples or no swelling during the solvent exchange process. The shrinkage value during the solvent exchange and drying processes is the highest for samples crosslinked with the highest radiation dose. It is hypothesized that the pectin contained in the sample undergoes degradation at high doses, resulting in a reduction in the material’s structural integrity [29,30]. Pectin degradation at lower doses occurs to a much lesser extent and is minimal. Consequently, the final product exhibits reduced shrinkage and an expanded specific surface area. Samples PVA/1 (27, 41, 58) kGy shrank significantly during drying under the supercritical carbon dioxide conditions that caused termination of investigations on sole PVA aerogels. These samples also contained ethanol after drying.

**Figure 4:**
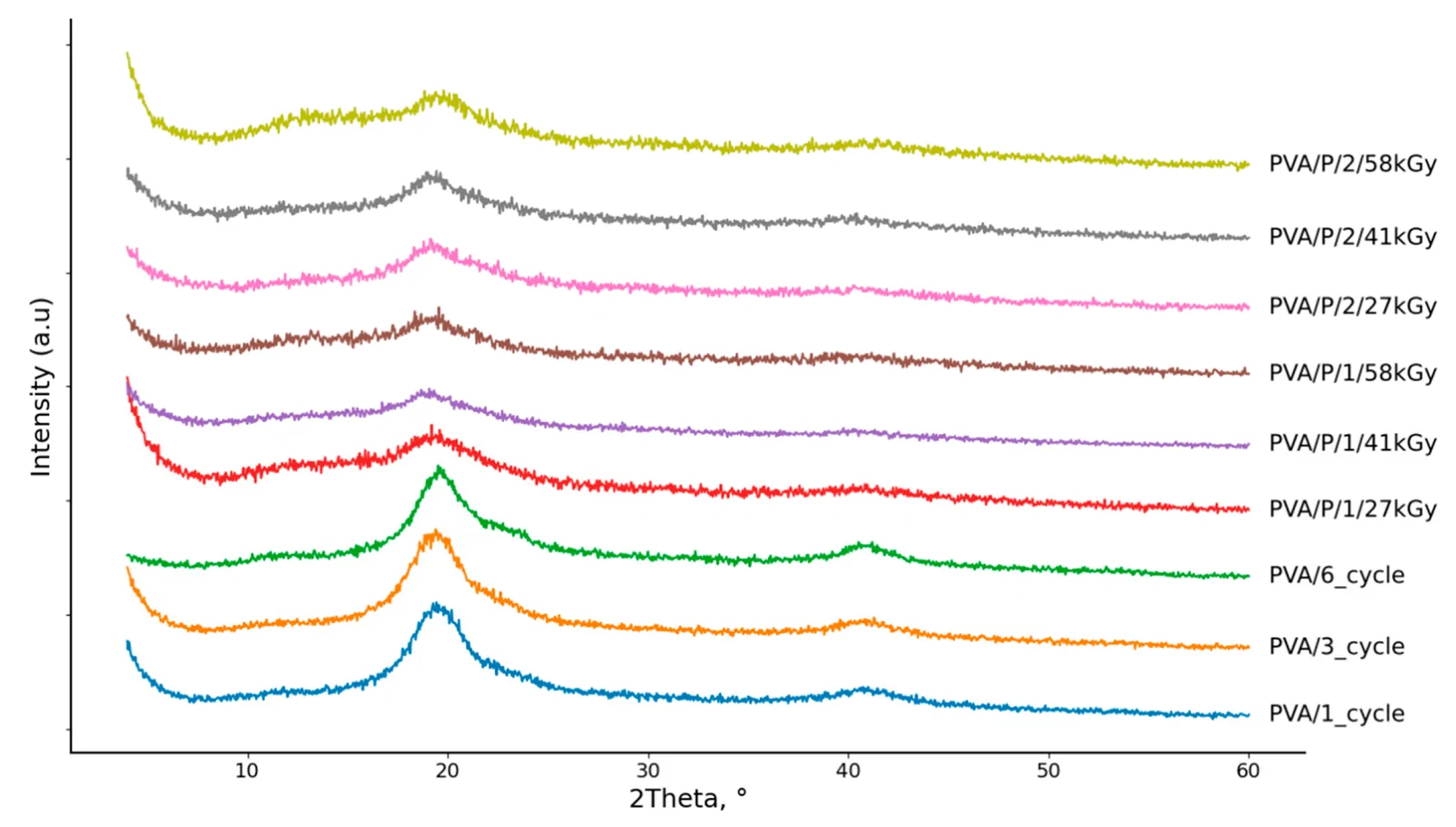
XRD diffraction patterns for aerogels after e-beam and freeze–thawing crosslinking.

XRD testing (Figure 4) support the explanation of shrinkage of the pure PVA sample during drying. As observed earlier [31], it was thought that the freeze–thaw cycles are the reason for crystalline structure formation. However, EB-induced crosslinking preserves the amorphous structure of the polymer [22]. Each diffractogram shows a characteristic peak at around 20°, which indicates the crystalline phase of PVA [32]. A clearly defined peak indicates partial crystallinity, while a broad, distorted band suggests an amorphous or fine crystalline structure. PVA samples crosslinked via freeze–thaw cycles exhibited sharper peaks. In contrast, samples crosslinked with radiation were characterized by wide, vague peaks. This is mainly due to the presence of pectin. Polysaccharide chains hinder the formation of PVA crystalline structures [33,34]. The crosslinking of PVA may result in a reduction in the long-range order in the polymer structure. A particular difference is visible for the highest dose of 58 kGy, where a drastic decrease in crystallinity is observed. This phenomenon can be attributed to an elevated number of crosslinks, which restrict the mobility of polymer segments and impede crystal formation.

### 2.3. Aerogel Chemistry

**Figure 5:**
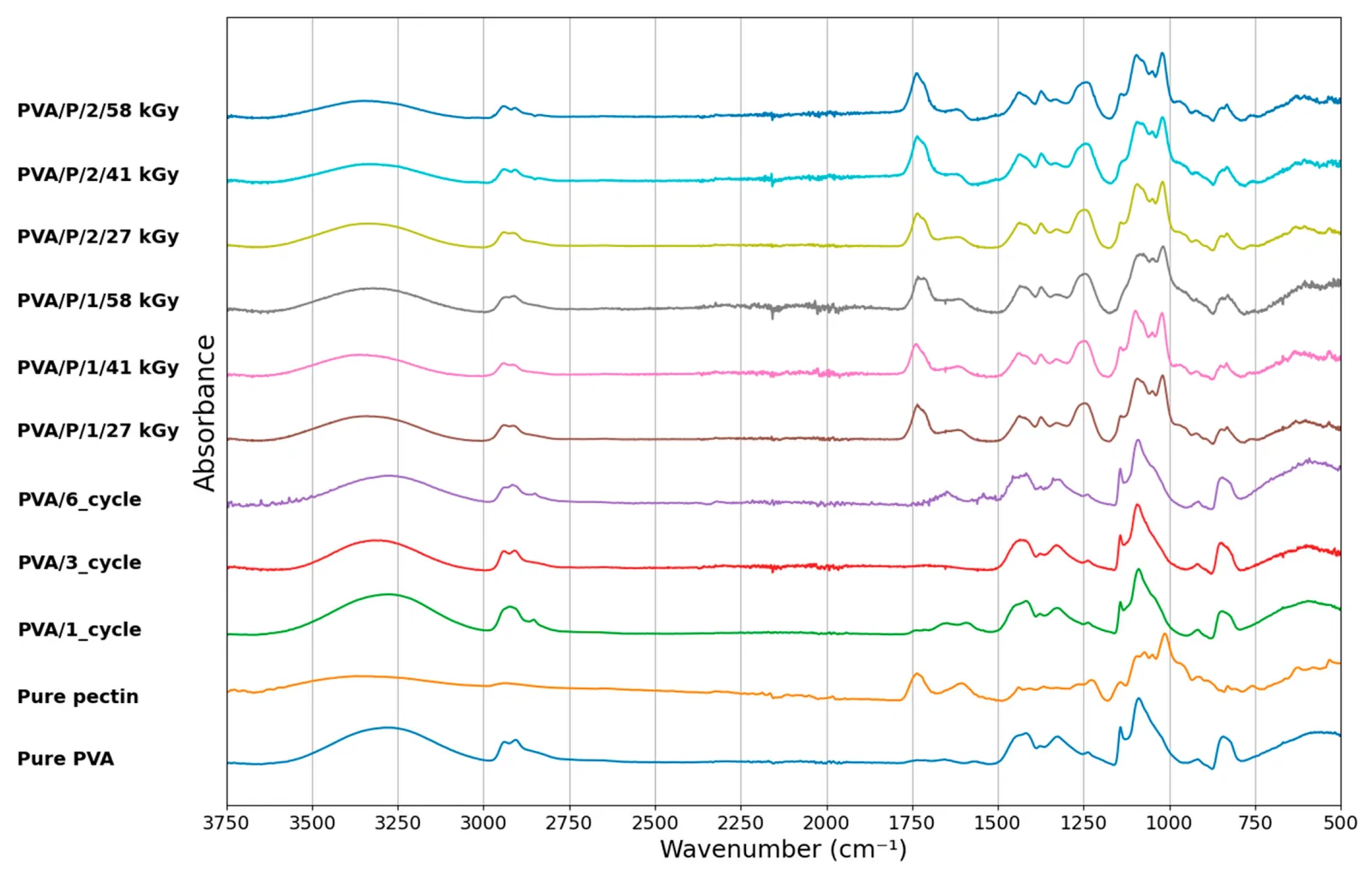
FT-IR spectra for crosslinked samples shown in the graph. Individual peaks are discussed in the text.

Figure 5 shows the absorption spectra of polymers forming aerogels manufactured via crosslinking with radiation and freeze–thaw methods. The spectra reveal bands characteristic for PVA and for pectin if it was present. Broad stretching vibrations of the O-H group occur at a wavelength of 3200–3400 cm^−1^. They originate from numerous hydroxyl groups of PVA and pectin, whereas stretching vibration at 2940–2880 cm^−1^ is associated with the C-H bonds present in PVA chains and pectin rings. Bands characterized for C=O groups at a wavelength of 1750–1700 cm^−1^ and bands at c.a. 1050 cm^−1^ of C-O are derived from pectin carboxyl groups [35]. This bond is absent in the polyvinyl alcohol spectrum. It has been demonstrated that the stretching vibrations of C–O–C and C–O bonds in the PVA matrix are particularly sensitive to the crystalline structure of PVA. The ~1140 cm^−1^ band is designated as the “crystallinity band” of PVA, and it has been established that the intensity of this band increases with higher crystallinity of the polymer [36]. PVA crosslinked via freeze–thaw cycles is characterized by a distinct band at this wavelength, which is indicative of ordered PVA segments; the intensity of this band increases with the number of the cycles. In contrast, the band at c.a. 1140 cm^−1^ is of reduced intensity in samples containing pectin. Moreover, this intensity undergoes a further decrease in proportion to the magnitude of the radiation dose. This FT-IR data finding indicates that electron beam crosslinking results in a decrease in the crystallinity of the sample. This provides additional confirmation for the XRD analysis. PVA hydrogels obtained using physical methods are structurally based on crystalline physical network knots (PVA crystals) and hydrogen bonds. It has been established that they do not form new covalent bonds; therefore, the FTIR spectrum of PVA/FT reflects pure poly(vinyl alcohol) with strong O–H bands and characteristic PVA bands (C–H, C–O) in the presence of water. On the other hand, irradiation results in the establishment of covalent bonds between chains. This occurs through the recombination of C-centered radicals on two distinct macromolecules, which were created upon the abstraction of H atoms. These types of bonds are primarily PVA–PVA, but may be potentially also PVA–pectin, as observed in other synthetic–natural polymer compositions exposed to irradiation [37]. The minimal number of crosslinking C-C bonds necessary to form the network involving all macromolecules is one per chain on average; therefore, such a minor change in functional groups, resulting from crosslinking, may not be revealed in the FT-IR spectra.

In the case of pectin, hydroxyl radicals (from the process of the radiolysis of water) readily abstract hydrogen atoms from the C1–C4 positions of the galacturonic acid, generating macroradicals localized on the polysaccharide backbone. Although radical formation is possible at all carbon atoms of the glacturonic acid residue, abstraction at the C5 position is substantially less probable, because this site is sterically and electronically protected by adjacent carboxyl or ester substituents [38]. Likewise, hydrogen abstraction from the methyl group of the ester functionality occurs with much lower probability, as consistently reported in the literature [39]. The macroradicals at the C1–C4 positions predominantly initiate degradation of the polysaccharide chain through cleavage of the β-(1-4)-glycosidic linkages connecting adjacent monosaccharide units. As a consequence, chain scission produces reactive chain-end radicals that are capable of participating in further radical reactions.

We hypothesize that the most likely mechanism for the crosslinking of pectins with PVA is that the pectin chains undergo radical-induced degradation, resulting in the formation of reactive radicals at their ends [40]. These radicals can either attach to the hydroxyl groups of PVA or recombine with other pectin chains. Pectin macromolecules can be covalently incorporated into the forming hydrogel network. Even though crosslinking reactions involving pectin are less efficient, the polysaccharide can form part of the gel. Yet, macroscopic changes of the crosslinked polymer are evident; for instance no crystalline phase is formed, and thus, the microstructure of the resulting aerogel is altered.

### 2.4. Thermal Stability

**Figure 6:**
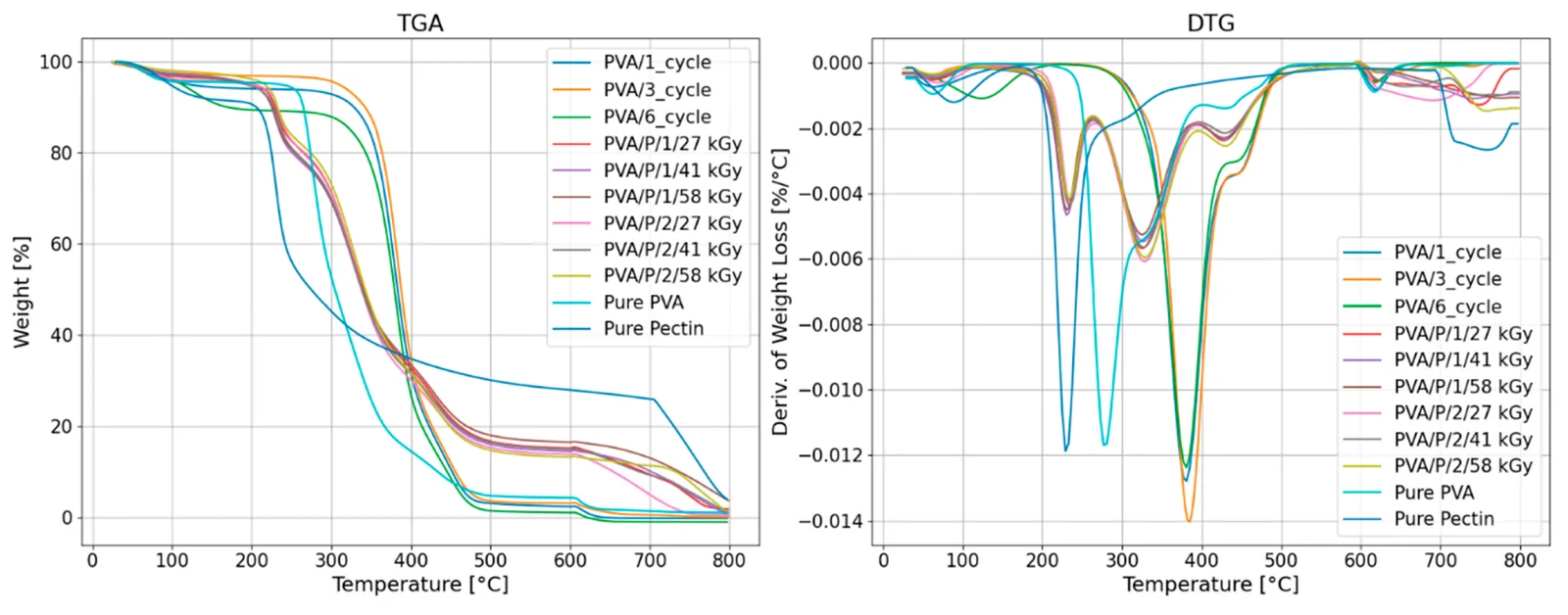
TGA profiles of aerogel samples prepared via freeze–thaw cycles (PVA) and crosslinked using an electron beam (PVA and pectin) compared to pure original components.

The thermogravimetric analysis is presented in Figure 6. In the initial phase, occurring within a temperature range of approximately 50–150 °C, the loss of water through evaporation is observed. The next stage occurs at a temperature of over 200 °C, marking the onset of pectin degradation and the commencement of PVA degradation. The principal decomposition of the pectin chain occurs at a temperature of approximately 200–300 °C, representing the continuation of the process initiated in stage II. The decomposition of the main PVA chain occurs in the range of approximately 250–350 °C. At these temperatures, the final breaking of C–C bonds in PVA chains and oxidation/dehydrogenation of residues takes place. It has been demonstrated that the amorphous state of a sample facilitates thermal degradation due to the absence of stabilizing crystallites—PVA and pectin samples. The most crystalline samples (PVA/FT) exhibited the highest decomposition temperatures and the slowest rate of weight loss, while the amorphous PVA/P composites began to decompose earlier [34,41].

The glass transition temperature (Tg) is a critical parameter in the context of supercritical drying, as it determines the thermal and mechanical stability of the gel network under drying conditions. As demonstrated in our recent studies on polylactic acid (PLA)-based gels, a significant reduction in Tg under CO_2_ pressure can lead to the collapse of the gel structure during supercritical drying. In the case of PLA, CO_2_ is known to act as a plasticizer, penetrating between polymer chains and increasing their molecular mobility. This results in a pronounced Tg depression, causing the material to soften or even melt inside the autoclave, thus making supercritical drying infeasible. Importantly, this phenomenon is driven primarily by the interaction between CO_2_ and the polymer matrix, rather than by hydrostatic pressure [42].

To confirm the statements regarding the change in the glass transition temperature of the samples using different crosslinking techniques, differential scanning calorimetry analysis was performed. The glass transition temperature of each sample is presented in Table 4 and on Figure S1 of the Supplementary Materials. Pure original PVA has a Tg of 77.7 °C, which is approximately 3–8 °C lower than that of samples crosslinked via freeze–thaw cycles. In contrast, radiation-crosslinked samples have a Tg approximately 20 °C higher. This confirms the effectiveness of radiation crosslinking. It also proves that no degradation of the PVA chain or pectin occurs during radiation crosslinking.

In our current study, we observed that the Tg (Table 4) of EB crosslinked pectin-based gels is approximately 15–20 °C higher than that of gels prepared via freeze–thaw cycling. This elevated Tg indicates a more rigid polymer network with reduced chain mobility, likely due to enhanced crosslink density and molecular packing. From a processing standpoint, such an increase in Tg suggests greater structural resilience during supercritical CO_2_ exposure. Consequently, these EB crosslinked gels are expected to better withstand the conditions in the autoclave without undergoing thermal collapse, thereby facilitating successful supercritical drying. In fact, this may explain why the textural properties of EB crosslinked aerogels are superior compared to aerogels obtained from the freezing–thawing process.

In semi-crystalline polymers such as PVA, an increase in crystallinity has been shown to limit the mobility of chains in the amorphous phase, resulting in a higher Tg [43]. Therefore, an XRD study was conducted to correlate the degree of crystallinity of resulting aerogels with the crosslinking technique. In general, an increase in the degree of crystallinity can lead to an increase in Tg because crystalline regions limit the mobility of polymer chains in amorphous regions [44].

### 2.5. Mechanical Strength of Aerogels

**Figure 7:**
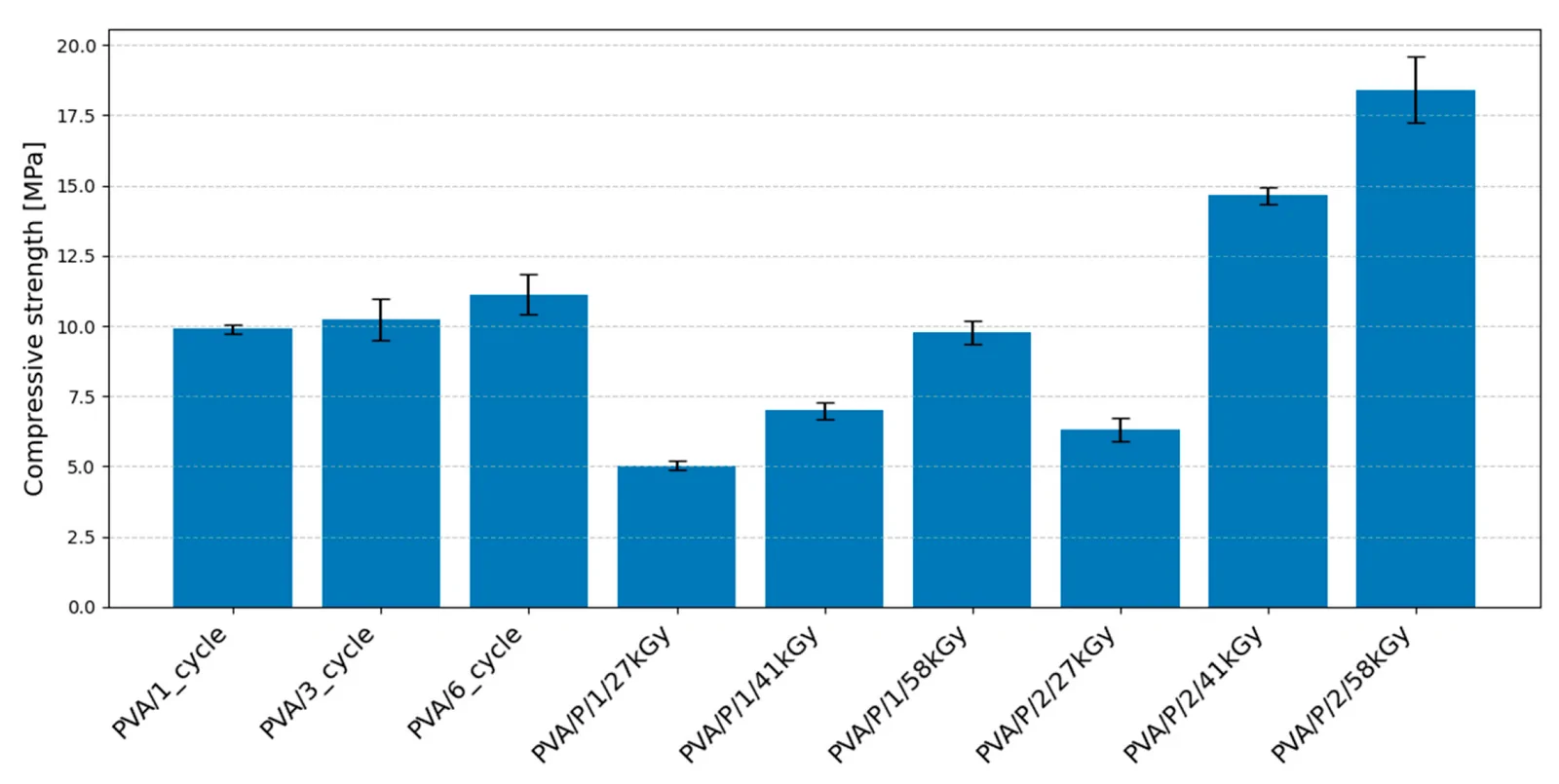
Compressive strength of the aerogel samples prepared via freeze–thaw cycles (PVA) and crosslinked using an electron beam (PVA and pectin).

In order to estimate the strength of aerogels, they were subjected to compression testing. Polyvinyl alcohol is an elastic polymer [43], and thus, the aerogel exhibits a deformation without rupture. Nevertheless, the material does not return to its original dimensions after the compression. When analyzing the results (Figure 7), it should first be noted that the concentration of PVA and pectin in the samples is an important factor. It is evident that an increase in the concentration results in a greater force being required to deform the sample. However, the effect of crosslinking processes on the strength of the samples is also significant. Samples preserved via freezing and thawing are characterized by a similar strength that slightly increases with the number of freezing and thawing cycles. Conversely, radiation-crosslinked samples exhibit a pronounced increase in the compressive strength, with respect to the increasing dose. A comparison of samples with analogous concentrations, such as PVA/1.3.6_cycle containing 10% PVA and PVA/P/2/27. The 41.58 kGy samples, which also have a comparable concentration of approximately 10%, reveal enhanced strength for higher doses of 41 or 58 kGy in comparison to samples that have undergone crosslinking via freeze–thaw cycles. This indicated that the application of radiation processing for preserving the hydrogel structure prior to drying may be advantageous with respect to the mechanical properties.

### 2.6. Aerogel Swelling

**Figure 8:**
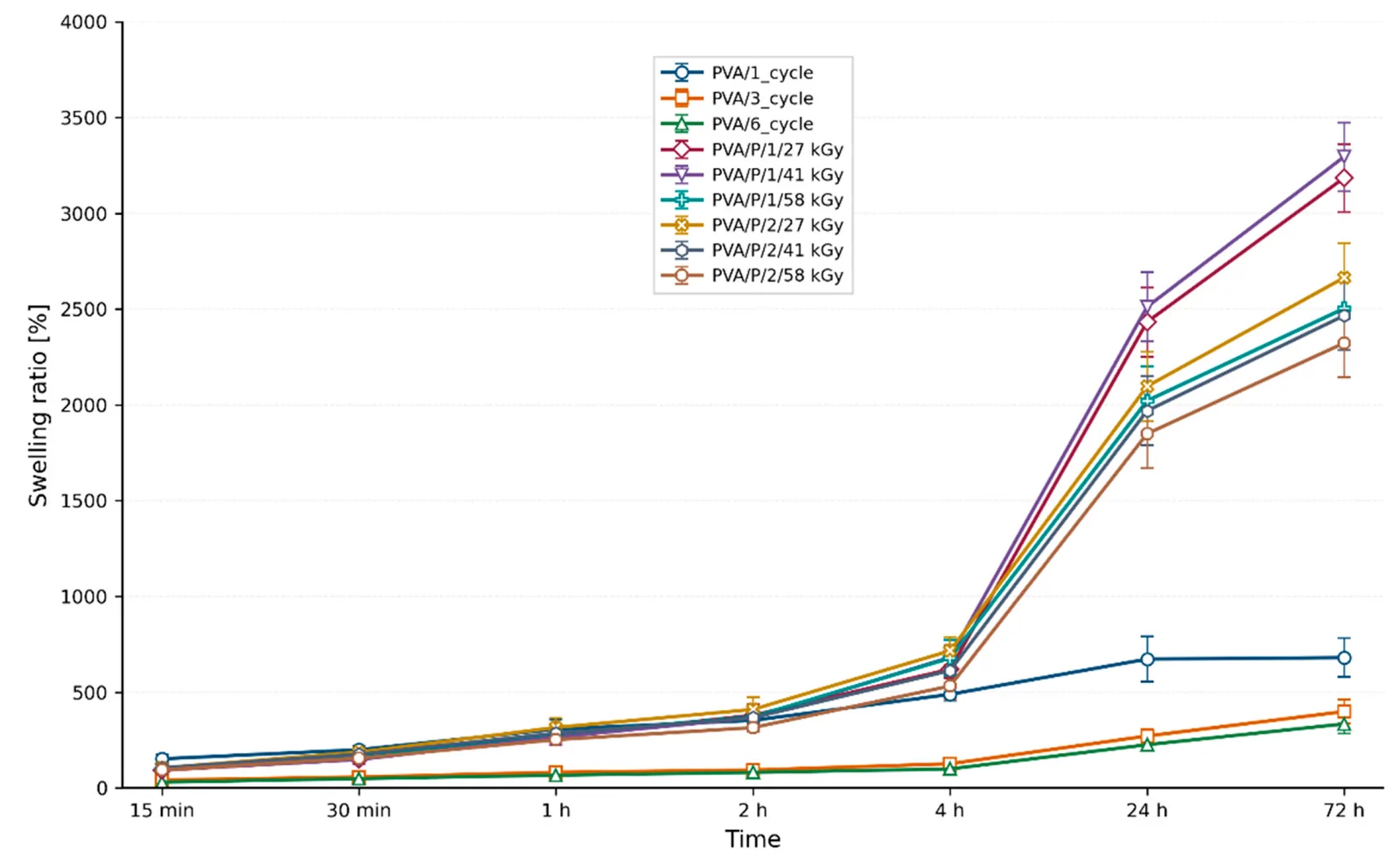
Swelling ratio of the aerogel samples prepared via freeze–thaw cycles (PVA) and crosslinked using an electron beam (PVA and pectin).

The behavior of the aerogel in water was assessed to determine its ability to absorb water whilst remaining stable. The samples were immersed in distilled water at a temperature of 25 °C for a period of 72 h. The aerogel’s water absorption capacity was expressed as a percentage of swelling. Upon immersion of the aerogel in water, a thin layer of hydrogel rapidly forms on its surface. As the aerogel absorbs water, this layer gradually thickens and the entire material swells, increasing in volume [45,46]. It was observed (Figure 8) that all electron-beam-crosslinked samples containing pectin exhibited an exceptionally high absorption capacity. In the case of PVA-only samples crosslinked via freeze–thaw cycles, swelling occurred much more slowly. PVA samples swell by approximately 400–500%. The degree of swelling is greatly influenced by the number of freeze–thaw cycles applied. A greater number of freeze–thaw cycles results in a denser PVA network, which limits the swelling of the aerogel. After 72 h of swelling, the sample subjected to one freeze–thaw cycle swells by approximately 650%, whereas samples subjected to three and six cycles swell by 400% and 330%, respectively. EB-crosslinked samples containing pectin swell to a much greater extent. These values range from 2500 to 3000%, which is due to the much higher specific surface area of aerogels made from pectin and PVA.

## 3. Discussion

**Figure 9:**
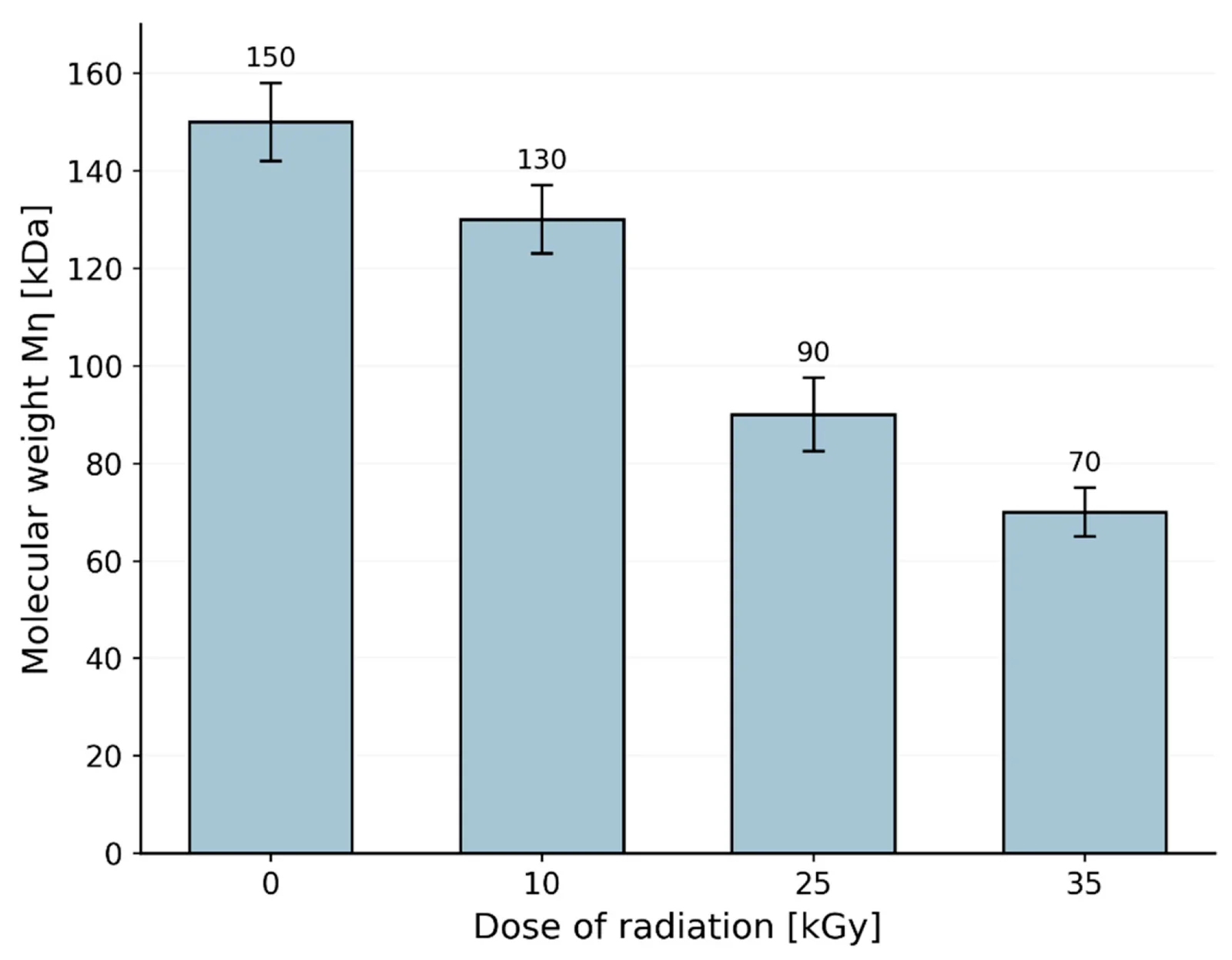
Viscosity-average molecular weight of pectin-powder (in solid state) subjected to ionizing radiation of accelerated electrons.

Studies were undertaken to determine whether it is possible to produce pectin and PVA hydrogels using radiation, which would then be dried in supercritical carbon dioxide to form an aerogel. Initially, irradiation tests were carried out on neat pectin, without any crosslinking agents or other polymers (P/1, P/2 samples). Figure 9 presents the results of the decrease in the molecular weight of pectin exposed, in the solid state, to irradiation doses of 10, 25, and 35 kGy. The observed degradation is mainly due to scission of the main chain, which is a typical degradation mechanism in polysaccharides. The reduction in the molecular weight is substantial because the pectin was irradiated in the solid form and therefore absorbed the entire radiation dose. In the case of the electron-beam irradiation of aqueous pectin or poly(vinyl alcohol) solutions, the radiation is absorbed by water, leading to water radiolysis producing transients that in turn produce radicals on macromolecules participating in polymer crosslinking therefore the formation of a polymeric network is possible.

It was demonstrated that achieving the crosslinking of pectin in aqueous solutions of low and moderate concentrations (<10%) requires the addition of a crosslinking agent. Consequently, avoiding a typical crosslinker, i.e., a molecule bearing unsaturated bonds, the most commonly being C=C, it was decided to use polyvinyl alcohol (PVA), which crosslinks if irradiated in an aqueous solution [10,11]. Although pectin degrades readily upon irradiation in dilute (0.5–5%) aqueous solutions, it was anticipated that the presence of PVA might influence the outcome of the radiation processing of these polymers. It was important to investigate the effect of the specific synthesis conditions used in this study on the formation of PVA–pectin hydrogels, as precursors of an aerogel.

The radical distribution among solutes results mainly from two factors, the ratio of concentrations of dissolved substances and the rate constants of transient water radiolysis products with a respective substance. For simple carbohydrates, as pectin is, and for carbon-based synthetic hydrophilic polymers, the main reacting species is hydroxyl radical. Hydrated electrons are scarcely reactive toward those compounds and a hydrogen atom is produced with a yield 5-fold lower than and reacts one or two orders of magnitude slower than the OH radical. As the rate constants of the OH radical with both polymeric components are similar [47], the concentrations of both may play a role—radicals on macromolecules will be distributed proportionally to their concentration ratio. Alpha hydroxyalkyl radicals are mainly created on PVA, whereas radicals at the beta carbon atom with respect to the hydroxyl group constitute not more than 30% of radicals [48]. Further, PVA undergoes crosslinking—the recombination of PVA macroradicals—and the pectin degrades. Therefore, pectin end-chain radicals formed upon scission may react with PVA macroradicals, yielding grafted molecules. It is an apparent reaction observed for combinations of poly(vinyl alcohol) and natural polymers irradiated in aqueous solution [40]. Whether the crosslinked structure is created depends on the PVA concentration, and its fraction should prevail over pectin.

The aerogel materials obtained using electron beam crosslinking exhibit bulk density values comparable to aerogels prepared using ionizing crosslinking methods. However, their specific surface area remains lower than that reported for pectin aerogels crosslinked with calcium ions, which typically exhibit values in the range of approximately 400–500 m^2^/g [16,17,47]. Nevertheless, the present results demonstrate that radiation-induced crosslinking represents an effective alternative strategy for producing highly porous polysaccharide-based aerogels without the need for chemical crosslinking agents.

The excessive shrinkage observed in samples of pure PVA subjected to electron beam irradiation might be attributed to the relatively high crosslinking density, as it was observed in the literature, prior drying under supercritical conditions. In the absence of pectin, radiation-induced crosslinking in aqueous PVA solutions at the doses used (27–58 kGy) appears to be very efficient, provoking contraction tensions. The tendency for contraction in crosslinked PVA is partially hindered by water in the hydrogel structure (bonded through hydrogen bonds and free filling the network), preventing collapse of the network; please note that polymer–water interactions are greater than polymer–polymer interactions in the PVA–water pair. However, upon exchange and removal of the solvent, a significant contraction occurs, as PVA tends to do.

This leads to significant network collapse and macroscopic shrinkage, rendering neat PVA samples unworthy of further detailed characterization. Based on our expertise and literature reports, high shrinkage results in a very poorly developed mesoporous structure, i.e., a low specific surface area, which does not allow the obtained material to be called an aerogel.

The addition of pectin likely contributes to network modification upon its formation through at least two mechanisms: (a) an increase in the overall polymer concentration in the gel matrix, (b) scission of bonds in the pectin mainchain, and therefore the potential formation of crosslinks between PVA and shortened pectin chains, yet not inducing contraction tensions; and (c) the presence of pectin prevents the material from forming a crystalline structure, namely PVA chains (see XRD data), and therefore maintains the material of a lower density and higher porosity at the nanoscale level (Table 2).

## 4. Materials and Methods

### 4.1. Materials

Poly(vinyl alcohol) (PVA) weight-average molecular weight 89–98 kDa and 99% hydrolyzed was purchased from Sigma Aldrich (St. Louis, MO, USA), and citrus pectin TCI (Tokyo, Japan) and ethanol 99.8% Avantor Performance Materials (Gliwice, Poland) were used as received. Purified water was used in all experiments. The pectin content in the citrus pectin reagent, determined via a thermogravimetric analysis (TGA), was found to be 8%, and in PVA, it was found to be 7.5%. Pectin is characterized by a galacturonic acid content of 83%, low methoxyl group level of 8%, and molecular weight of 1.49 · 10^5^ g·mol^−1^.

### 4.2. Preparation of Pectin and Pectin/PVA Aerogels

**Figure 10:**
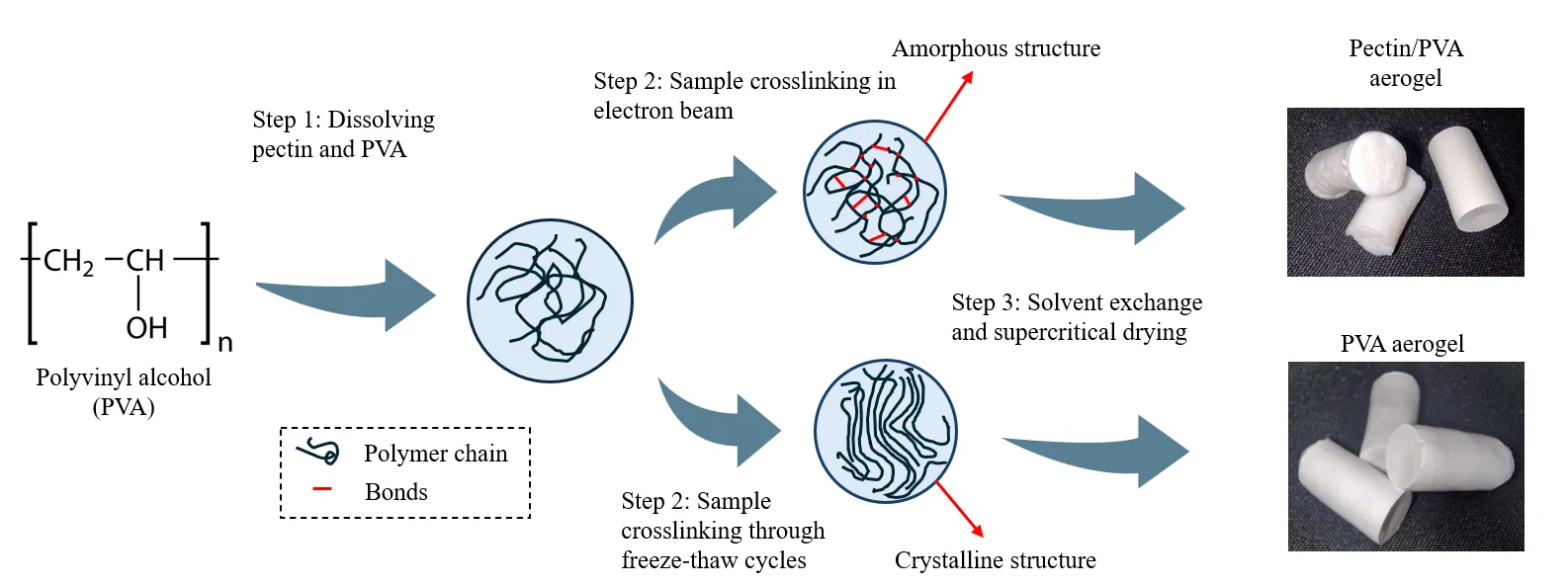
The process of preparing aerogels using electron beam and freeze–thaw techniques to produce stable hydrogels. Step 1: Dissolve PVA or PVA/pectin composite in water. Step 2: Crosslink the samples via irradiation or freeze–thaw cycles. Step 3: Replace the solvent with ethanol. Each image shows the samples obtained in comparison with the samples with the lowest shrinkage denoted as the PVA aerogel.

Figure 10 illustrates the procedure for the synthesis of aerogels. The study involved the preparation of aerogels based on pectin, poly(vinyl alcohol), and pectin/poly(vinyl alcohol) composites. Two crosslinking techniques were employed, each suitable for different formulations: irradiation-induced crosslinking and freeze–thaw cycles.

#### 4.2.1. Aerogel Preparation

Polyvinyl alcohol hydrogel samples were prepared by dissolving PVA in water at 80 °C for 3 h with stirring (250 rpm). The same procedure was followed for the dissolution of pectin with polyvinyl alcohol. The compositions of PVA samples are presented in Table 5. The pH of the pectin solution was c.a. 5, the pH of the PVA solution was c.a. 8, and the pH of the pectin/PVA solution was c.a. 7.

The solutions were then poured into syringes and sealed with Parafilm^®^. Ten samples of each type were prepared. Two methods were used for gelling or solidification: freeze–thawing cycles and EB irradiation. The process of freeze–thawing consisted of placing the samples in a freezer at −20 °C for 24 h and then storing the samples at room temperature for one hour. Based on previous studies [32], the degree of crystallinity increases with the number of freeze/thaw cycles, reaching a plateau at 5 cycles. Therefore, one, three, and six freeze/thaw cycles were performed. Alternatively, an EB of 6 MeV energy generated from a linac machine (ELU-6) was used for crosslinking. Irradiation of the PVA polymer in an aqueous solution was performed in a semi-continuous mode with a dose rate of ca. 5 kGy/min, to three total doses of 27, 41, and 58 kGy. Alanine and radiochromic film dosimetries were used to determine the dose rate. The obtained hydrogel cylinders were of 30 mm and 10 mm in the length and diameter, respectively. The sample formulations were prepared taking into account the water content present in the pectin and PVA, by adjusting the amount of water added during the dissolution of the ingredients accordingly.

#### 4.2.2. Solvent Exchange

For the preparation of hydrogels for drying, the solvent was replaced through continuous elution with ethanol solutions in consecutive increasing concentrations of 20, 40, 60, and 80% at room temperature. In the final step, the samples were rinsed four times with ethanol. To determine the duration of each solvent exchange step, the ethanol concentration was controlled by a density meter (DMA 4500, Anton Paar Company, Graz, Austria) at regular intervals. This made it possible to determine the optimum time after which the next exchange step could be performed. Two hours was sufficient to completely replace the solvent in a sample. When the final ethanol concentration reached 98%, the samples were transferred to supercritical CO_2_ for drying.

#### 4.2.3. Supercritical Drying

In order to minimize sample shrinkage, drying was carried out under supercritical carbon dioxide conditions. In this process, the ethanol in the sample is replaced by carbon dioxide. The drying process was carried out in the following steps: (a) compression of liquid carbon dioxide in an autoclave until 120 bar, (b) the dynamic phase of the continuous flow of liquid carbon dioxide at a rate of 40 mL/min, (c) the liquid CO_2_ preheated to a 40 °C flow into the sample chamber, and (d) controlled depressurization of the system at a rate of 5 bar/min in order to prevent the sample structure from collapsing. The entire drying process takes ca. 2 h.

Supercritical CO_2_ drying is a well-established technique in aerogel synthesis due to its ability to eliminate surface tension at the liquid–gas interface, thus preserving the porous microstructure. The selected parameters (pressure, temperature, and flow rate) are within the typical range for the effective drying of PVA-based gels. The depressurization rate of 5 bar/min is conservative enough to minimize mechanical stress during the phase transition from supercritical to gaseous CO_2_, further supporting the structural integrity.

### 4.3. Measurements

The shrinkage and bulk density of the aerogel was calculated (1) by taking volume measurements with a caliper with a measurement accuracy of 0.03 mm, and the sample mass was determined with an analytical balance with an accuracy of 0.001 g. The shrinkage was calculated both for the solvent exchange process and during the drying process in supercritical carbon dioxide conditions. Three replicates of the tests were carried out for each sample, and the standard deviation is reported.

(1) $$Volumetric\;shrinkage=\frac{V_{A}-V_{H}}{V_{A}}\cdot 100$$

where $V_{A}$ is the volume of the aerogel, and $V_{H}$ is the volume of the hydrogel.

To determine the presence of crystalline phases, an X-ray diffraction (XRD) experiment was performed using an X-ray diffractometer, Bruker model D2 Phaser (Billerica, MA, USA), equipped with a Lynxeye detector (Billerica, MA, USA).

Low-temperature nitrogen adsorption–desorption physisorption measurements were performed to characterize the microstructural properties of supercritically dried samples (Nova 3000e Surface Area Analyzer, Quantachrome Instruments, Boynton Beach, FL, USA). Prior to analysis, each sample was degassed under a vacuum at 60 °C for at least 6 h in the measurement cell. Approximately 20–30 mg of material was used per measurement. The mass-specific surface area was determined using the Brunauer–Emmett–Teller (BET) method within a relative pressure range (p/p_o_) of 0.027–0.27. The specific mesopore volume and average mesopore diameter were calculated using the Barrett–Joyner–Halenda (BJH) method. Three replicates of the tests were carried out for each sample, and the standard deviation is reported.

Total porosity of the aerogels was estimated (2) based on the bulk density and skeletal density according to the Equation:

(2) $$\varphi =\left(1-\frac{\rho_{b}}{\rho_{s}}\right)\times 100$$

where $\rho_{s}$ is the skeletal density of the polymer matrix, and bulk density ($\rho_{b}$) was calculated from the measured mass-to-volume ratio of dried aerogel samples. Skeletal density ($\rho_{s}$) values were estimated based on literature data for the neat polymers and calculated using the rule of mixtures for composite formulations. The skeletal density of poly(vinyl alcohol) (PVA) was assumed to be 1.19 g/cm^3^ according to supplier specifications for highly hydrolyzed PVA, whereas the density of pectin was taken as 1.50 g/cm^3^ according to literature reports for pectin [17]. Three replicates of the tests were carried out for each sample, and the standard deviation is reported. For pectin/PVA composites, the skeletal density was estimated (3) from the mass fractions of individual components using the rule of mixtures.

(3) $$\rho_{s}={\left(\frac{w_{PVA}}{\rho_{PVA}}+\frac{w_{pectin}}{\rho_{pectin}}\right)}^{-1}$$

where *w* represents the weight fraction of each component in the polymer matrix.

To determine the chemical bonds formed during the gelation process, Fourier transform infrared (FT-IR) spectroscopy was used. FT-IR absorption spectra were obtained using a Nicolet 6700 FT-IR (Thermo Scientific, Waltham, MA, USA) spectrometer with Smart Orbit ATR diamond sampling equipment in the range of 4000–400 cm^−1^. The number of scans used was 64 with a resolution of 4 cm^−1^.

The morphology of the aerogels was determined based on images of the pore structure taken with a JSM-5500 LV scanning electron microscope (JEOL Ltd., Tokyo, Japan). All microscopic observations were made in high-vacuum mode and at an accelerating voltage of 10 kV. Samples were coated with gold before observations.

TGA was used to investigate the thermal properties of aerogels with a thermogravimetric/differential scanning calorimeter, TGA/DSC1 (Mettler Toledo, Greifensee, Switzerland). The aerogel samples of a c.a. 10 mg initial weight were heated in aluminum pans from 25 °C to 900 °C at a rate of 10 °C/min. The measurements were conducted within the temperature range of 25 °C to 600 °C, in an argon atmosphere with a flow rate of 50 mL/min. Subsequently, the gas was replaced with air (50 mL/min), and the heating was continued to a temperature of 900 °C. One replicate of the tests was carried out for each sample.

A differential scanning calorimeter (DSC1, Mettler Toledo, Greifensee, Switzerland) was employed to determine the glass transition temperature of the samples. Aerogels with a mass of approximately 10 mg were heated from 25 °C to 270 °C in an argon atmosphere at a heating rate of 10 °C/min. One replicate of the tests was carried out for each sample.

To examine the compressive mechanical strength of the aerogel samples, compression testing was undertaken using a Zwick Z100 testing machine (Zwick/Roell Group, Ulm, Germany) with a load cell of 2 kN and a speed of 2 mm min^−1^. The cylinder samples with thicknesses of 15 mm were compressed with a flat compression plate with a 50 mm diameter. The measurements were conducted at 25 °C and 40% relative humidity. The test was performed on 6 samples for each series, and the standard deviation is reported.

The viscosity of pectins [η] was determined using viscometry and the Huggins method, employing a type I Ubbelohde capillary viscometer with a capillary diameter of 0.63 mm. The solvent was a 0.1 M NaCl solution, and the measurements were carried out at 25 °C. The molecular weight Mη was calculated (4) using the Mark–Houwink equation: it can be demonstrated that

(4) $$\left[\eta \right]=KM_{\eta }^{\alpha }$$

where *K* = 2.34 · 10^−4^ and *α* = 0.822 [49]. The molecular weight of pectin was determined for initial pectin and samples irradiated with doses of 10, 25, and 35 kGy in order to confirm the degradation of pectin. Three replicates of the tests were carried out for each sample, and the standard deviation is reported.

To determine the swelling capacity, the aerogels were cut into smaller pieces, weighed (0.01 g), and immersed in test tubes containing distilled water (30 mL) at room temperature (25 ± 0.5 °C). At set intervals (15, 30 min; 1, 2, 4, 24, 72 h), the samples were removed, carefully dried with laboratory paper to remove excess liquid, and then weighed on an analytical balance. The swelling coefficient was calculated using Equation (5):

(5) $$Swelling\;ratio\left(\%\right)=\frac{m_{WET}-m_{DRY}}{m_{DRY}}\cdot 100$$

where $m_{WET}$ is the wet mass of the aerogels at time *t*, and $m_{DRY}$ is the initial dry mass of the aerogels. After weighing, the samples were returned to the vials. Three replicates of the tests were carried out for each sample, and the standard deviation is reported.

## 5. Conclusions

The findings of the conducted research demonstrated that the radiation-induced crosslinking of the pectin/PVA hydrogel system constitutes a viable approach for drying under supercritical conditions in order to control the structure of a resulting aerogel. The presence of pectin limits the shrinkage of the material during the drying process, while also increasing of the glass transition temperature, preventing the gels from collapse during the drying. It was demonstrated that the electron beam crosslinking of pectin/PVA in aqueous solution is a method that allows tailoring the properties of the final aerogel material. This technique ensures better structural integrity and dimensional stability of the aerogel, which is especially critical during solvent exchange and supercritical CO_2_ drying. Furthermore, the combination of pectin and radiation crosslinking resulted in an increased specific surface area and reduced macroporosity, enhancing the material’s mechanical characteristics. These findings indicate that such aerogels could be promising candidates for use in applications requiring lightweight, porous materials with tailored porosity and surface properties, such as thermal insulation, controlled release, or environmental remediation. Upon successful testing, the materials presented can be successfully used as wound dressings due to their ability to absorb large amounts of liquid (2000% by weight). As noted in the experimental section, it is possible to control the absorption properties of aerogels by adjusting their porosity, selecting materials based on their hydrophilic properties, or altering the weight ratios of those already used. In this way, it is possible to create a wound dressing suitable for wounds with heavy exudate; such a material should not disintegrate at the site of application, as demonstrated in the presented studies. However, to confirm this hypothesis, future studies should be conducted in fluids mimicking the wound environment, as well as future animal tests.

Furthermore, despite the meaningful results of laboratory-scale experiments, challenges remain in scaling up the production of aerogels. To address these challenges, cost-effectiveness, process repeatability, and safety must be carefully optimized during industrial implementation to ensure commercial viability. Parameters such as the drying time, homogeneity of crosslinking, and energy efficiency are critical for industrial feasibility and require further investigation. Overall, this study provides new insights into the properties of pectin and pectin/PVA aerogels obtained with radiation and demonstrates the potential of these materials for further development.

## 6. Patents

Patent application: Title: “Sposób sieciowania kompozycji pektyna-poli(alkohol winylowy) wiązką elektronów w celu otrzymania porowatych materiałów aerożelowych.” Number: P.456077 [WIPO ST 10/C PL456077].

## Figures and Tables

**Table 1:** Observations on gel formation.

Sample Name	Observations
PVA/1_cycle	Gel stable
PVA/3_cycle	Gel stable
PVA/6_cycle	Gel stable
P/1 (27, 41, 58) kGy	Gel absent
P/2 (27, 41, 58) kGy	Gel absent
PVA/P/1 (27, 41, 58) kGy	Gel stable
PVA/P/2 (27, 41, 58) kGy	Gel stable
PVA/P/3 (27, 41, 58) kGy	Gel absent
PVA/P/4 (27, 41, 58) kGy	Gel fragmentation during solvent exchange
PVA/1 (27, 41, 58) kGy	Gel stable

**Table 2:** Physical characterization of aerogels of PVA crosslinked via freeze–thawing (FT) and a composite of PVA and pectin crosslinked using an electron beam (irradiation dose indicated in the sample name). The standard deviation of the measurements is estimated to be ±20 m^2^/g based on our previous measurements of various aerogel samples.

Sample Name	Surface Area BET [m^2^/g]	Average Pore Diameter [nm]	*ρ*_b_ [g/cm^3^] n = 3	*ρ*_s_ [g/cm^3^]	φ [%]
PVA/1_cycle	46	19	0.22 ± 0.04	1.19	81.5
PVA/3_cycle	34	17			
PVA/6_cycle	28	18			
PVA/P/1/27 kGy	238	29	0.11 ± 0.02	1.27	91.3
PVA/P/1/41 kGy	237	23			
PVA/P/1/58 kGy	203	19			
PVA/P/2/27 kGy	241	27	0.10 ± 0.02	1.27	92.3
PVA/P/2/41 kGy	200	23			
PVA/P/2/58 kGy	212	22			

**Table 3:** Percentage of shrinkage of the gel after solvent exchange and for aerogels after supercritical drying.

Sample Name	Shrinkage During Solvent Exchange [%]	Shrinkage During Supercritical Drying [%]	Total Shrinkage [%]
PVA/1_cycle	0.7 ± 0.2	31 ± 2	31 ± 2
PVA/3_cycle	1.4 ± 0.5	30.3 ± 0.2	31.7 ± 0.7
PVA/6_cycle	1.6 ± 0.3	30.1 ± 0.2	31.7 ± 0.5
PVA/P/1/27 kGy	2.6 ± 0.9	36 ± 1	39 ± 2
PVA/P/1/41 kGy	4.0 ± 0.5	44.4 ± 0.4	48.5 ± 0.9
PVA/P/1/58 kGy	5.3 ± 0.2	47 ± 1	53 ± 2
PVA/P/2/27 kGy	0.7 ± 0.2	35 ± 1	36 ± 2
PVA/P/2/41 kGy	2 ± 1	34 ± 2	36 ± 3
PVA/P/2/58 kGy	9.5 ± 0.9	38.3 ± 0.2	48 ± 1
PVA/1/27 kGy	2.1 ± 0.2	60.1 ± 0.7	62.2 ± 0.9
PVA/1/41 kGy	2.5 ± 0.3	66.3 ± 0.4	68.8 ± 0.8
PVA/1/58 kGy	2.3 ± 0.2	68.1 ± 0.6	71 ± 0.3

**Table 4:** Glass transition temperatures of PVA in aerogel samples prepared via freeze–thaw cycles (PVA) and crosslinked using an electron beam (PVA and pectin) compared to pure original components, as determined via differential scanning calorimetry.

Sample Name	Tg [°C]
PVA/1_cycle	80.5
PVA/3_cycle	81.7
PVA/6_cycle	85.2
PVA/P/1/27 kGy	101.8
PVA/P/1/41 kGy	97.5
PVA/P/1/58 kGy	102.5
PVA/P/2/27 kGy	103.3
PVA/P/2/41 kGy	102.1
PVA/P/2/58 kGy	95.4
Pure PVA	77.7
Pure Pectin	-

**Table 5:** The composition of individual samples. P/1, P/2, PVA/P/1, PVA/P/2, PVA/P/3, or PVA/P/4 was prepared with EB-crosslinking, and they are named with radiation doses (27, 41, 58 kGy) in sequence. Samples PVA/1_cycle, PVA/3_cycle, and PVA/6_cycle were treated with freeze–thaw cycles.

	Weight Parts		
Sample Name	Pectin	PVA	Water
PVA/1_cycle	-	10	100
PVA/3_cycle	-	10	100
PVA/6_cycle	-	10	100
P/1 (27, 41, 58) kGy	5	-	100
P/2 (27, 41, 58) kGy	7	-	100
PVA/P/1 (27, 41, 58) kGy	2	5	100
PVA/P/2 (27, 41, 58) kGy	2.8	7	100
PVA/P/3 (27, 41, 58) kGy	5	2	100
PVA/P/4 (27, 41, 58) kGy	7	2.8	100
PVA/1 (27, 41, 58) kGy	-	10	100

## Data Availability

The raw data supporting the conclusions of this article will be made available by the authors on request.

## Supplementary Materials

The following supporting information can be downloaded at: https://www.mdpi.com/article/10.3390/ijms27188047/s1.

## Abbreviations

The following abbreviations are used in this manuscript:

PVA	poly(vinyl alcohol)
EB	electron beam
