# Supplementary material for: Electron Beam Crosslinking as a Strategy for the Synthesis of Pectin/PVA Aerogels: Effect of Concentration and Radiation Dose
Source: Int J Mol Sci. 2026 Sep 10;27(18):8047. doi: 10.3390/ijms27188047 (PMC13607514; doi:10.3390/ijms27188047)
Supplement: Supplementary file 1 [file ijms-27-08047-s001.zip › ijms-4466199-supplementary.pdf]

Supplementary Material

Electron beam cross-linking as a strategy for the synthesis of pectin/PVA aerogels: effect of concentration and radiation dose

Dawid Szymborski [1,*], Radosław Wach [2], Pavel Gurikov [3, 4, 5], Anna Strąkowska [1], Krzysztof Strzelec [1], Maher Saifi [6], Renata Boris [7]

[1] Institute of Polymer and Dye Technology, Faculty of Chemistry, Lodz University of Technology, Stefanowskiego 16, 90-537 Lodz, Poland. E-mail: dawid.szymborski@dokt.p.lodz.pl

[2] Institute of Applied Radiation Chemistry, Faculty of Chemistry, Lodz University of Technology, Wroblewskiego 15, 93-590 Lodz, Poland

[3] Institute of Thermal Separation Processes, Hamburg University of Technology, Eißendorfer Straße 38, 21073 Hamburg, Germany

[4] aerogel-it GmbH, Albert-Einstein-Str. 1, 49076 Osnabrück, Germany

[5] United Nations University Hub on Engineering to Face Climate Change at the Hamburg University of Technology, United Nations University Institute for Water, Environment and Health (UNU-INWEH), Hamburg, Germany

[6] Aix Marseille Univ, CNRS, Centrale Marseille, M2P2, Marseille, France

[7] Laboratory of Composite Materials, Institute of Building Materials, Faculty of Civil Engineering, Vilnius Gediminas Technical University, Linkmenų St. 28, Vilnius 08217, Lithuania

* Correspondence: dawid.szymborski@dokt.p.lodz.pl

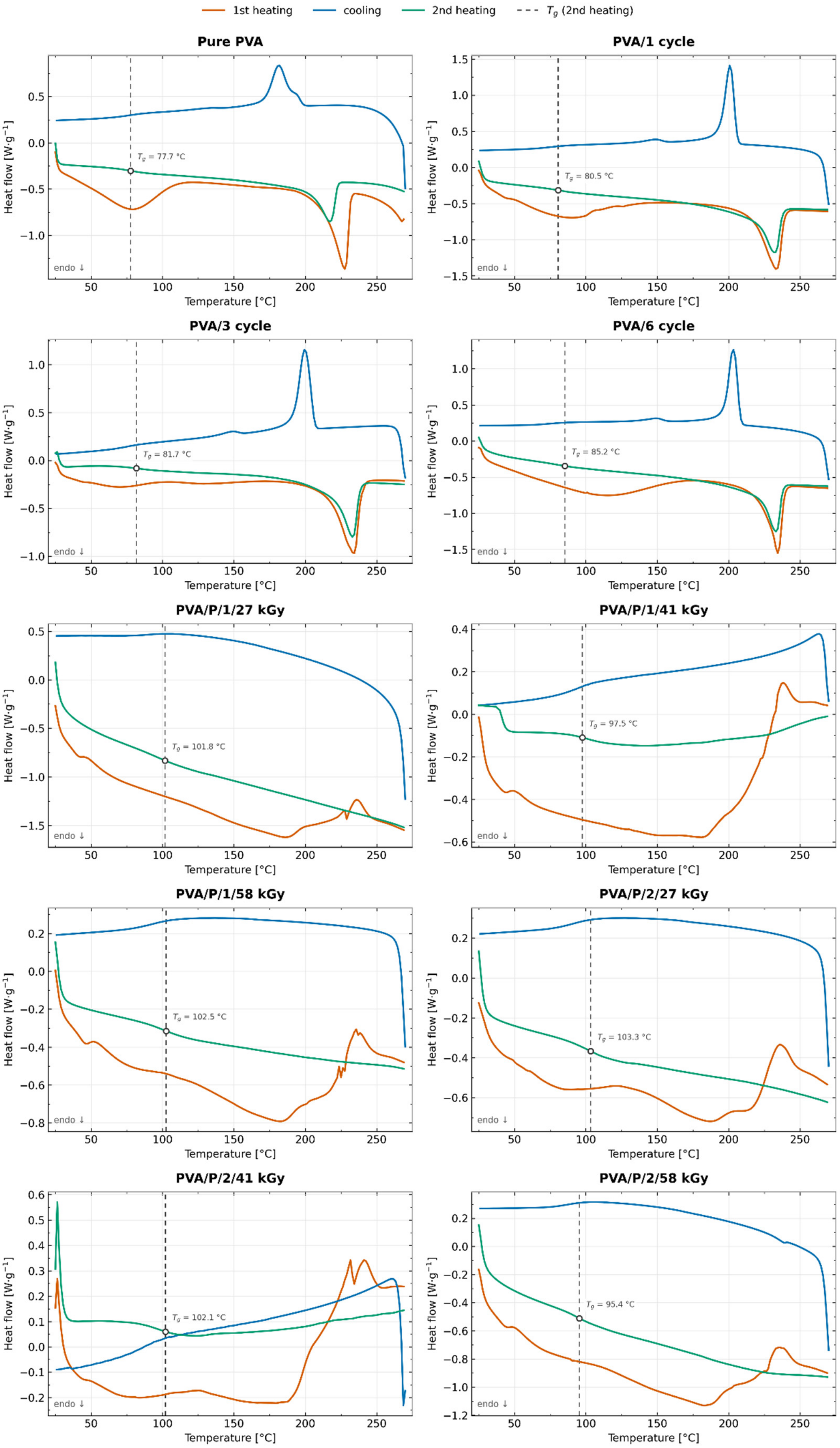

Figure S1. DSC profiles of aerogel samples prepared by freeze-thaw cycles (PVA) and crosslinked by electron beam (PVA and pectin) compared to pure original components.
